# Dose-to-Duration Encoding and Signaling beyond Saturation in Intracellular Signaling Networks

**DOI:** 10.1371/journal.pcbi.1000197

**Published:** 2008-10-10

**Authors:** Marcelo Behar, Nan Hao, Henrik G. Dohlman, Timothy C. Elston

**Affiliations:** 1 Department of Physics, University of North Carolina Chapel Hill, Chapel Hill, North Carolina, United States of America; 2 Program in Cellular and Molecular Biophysics, University of North Carolina Chapel Hill, Chapel Hill, North Carolina, United States of America; 3 Department of Pharmacology, University of North Carolina Chapel Hill, Chapel Hill, North Carolina, United States of America; 4 Department of Biochemistry and Biophysics, University of North Carolina Chapel Hill, Chapel Hill, North Carolina, United States of America; UT Southwestern Medical Center, United States of America

## Abstract

The cellular response elicited by an environmental cue typically varies with the strength of the stimulus. For example, in the yeast *Saccharomyces cerevisiae*, the concentration of mating pheromone determines whether cells undergo vegetative growth, chemotropic growth, or mating. This implies that the signaling pathways responsible for detecting the stimulus and initiating a response must transmit quantitative information about the intensity of the signal. Our previous experimental results suggest that yeast encode pheromone concentration as the duration of the transmitted signal. Here we use mathematical modeling to analyze possible biochemical mechanisms for performing this “dose-to-duration” conversion. We demonstrate that modulation of signal duration increases the range of stimulus concentrations for which dose-dependent responses are possible; this increased dynamic range produces the counterintuitive result of “signaling beyond saturation” in which dose-dependent responses are still possible after apparent saturation of the receptors. We propose a mechanism for dose-to-duration encoding in the yeast pheromone pathway that is consistent with current experimental observations. Most previous investigations of information processing by signaling pathways have focused on amplitude encoding without considering temporal aspects of signal transduction. Here we demonstrate that dose-to-duration encoding provides cells with an alternative mechanism for processing and transmitting quantitative information about their surrounding environment. The ability of signaling pathways to convert stimulus strength into signal duration results directly from the nonlinear nature of these systems and emphasizes the importance of considering the dynamic properties of signaling pathways when characterizing their behavior. Understanding how signaling pathways encode and transmit quantitative information about the external environment will not only deepen our understanding of these systems but also provide insight into how to reestablish proper function of pathways that have become dysregulated by disease.

## Author Summary

Cells must be able to detect and respond to changes in their surroundings. Often environmental cues, such as hormones or growth factors, are received by membrane receptors that in turn activate intracellular signaling pathways. These pathways then transmit information about the stimulus to the cellular components required to elicit an appropriate response. In many cases, the nature of the response depends on the dose of the stimulus. Thus, in addition to relaying qualitative information (e.g., the presence or absence of a stimulus), signaling pathways must also transmit quantitative information about the intensity of the stimulus. Here we introduce “dose-to-duration” encoding as an effective strategy for relaying such information. We demonstrate that by providing a mechanism for overcoming saturation effects, modulation of signal duration increases the range of stimulus concentrations for which dose-dependent responses are possible. This increased dynamic range produces the counterintuitive result of “signaling beyond saturation” in which dose-dependent responses are still possible after apparent saturation of the receptors. Finally, we demonstrate that dose-to-duration encoding is used in the yeast mating response pathway and presents a simple mechanism that can account for current experimental observations.

## Introduction

Many substances, such as hormones, neurotransmitters and a variety of pharmaceuticals, affect cellular behavior by binding to membrane receptors and activating intracellular signaling pathways. These pathways transmit information from the plasma membrane to selected cellular components to generate an appropriate response to the environmental cue. However, signaling networks are not simply passive relay systems, but actively modulate the transmitted signals. For example, cross inhibition is used to avoid spurious crosstalk between pathways. Similarly, negative feedback allows pathways to adapt or desensitize to persistent stimuli [1],[2]. In many cases, the nature of the response depends on the dose of the stimulus. Thus, in addition to relaying qualitative information (e.g. the presence or absence of a stimulus), signaling pathways must also transmit quantitative information about the intensity of the stimulus.

Many signaling pathways consist of a cell surface receptor, G protein transducer, and a series of protein kinases, including a mitogen activated protein kinase (MAPK). This architecture is widely employed in mammalian cells, but is also found in single-cell eukaryotes such as yeast [3]. The pheromone response pathway of the yeast *Saccharomyces cerevisiae* provides an instructive example in which the elicited cellular response depends on the concentration of the stimulus. At low pheromone levels, cells continue vegetative growth. At intermediate concentrations, cells develop an elongated morphology and in the presence of a pheromone gradient the growth is directed to the source of the stimulus, a process known as chemotropic growth [4]–[7]. Finally, at high pheromone concentrations cells initiate a mating program that eventually leads to growth arrest and the development of mating projections (for a review see [3]). Therefore, for yeast to make the correct developmental decision, quantitative information about the pheromone concentration must be reliably transmitted to the appropriate cellular components. Here we use mathematical modeling to investigate the different ways this information can be transferred. The results of this analysis taken together with our recently published data demonstrate that the pheromone pathway uses a strategy in which the agonist dose is encoded as the duration of the signal. Because the yeast pheromone response pathway consists of a G-protein coupled receptor and MAP kinase cascade, the results of our investigations should have direct implications for signal transduction in mammalian cells.

## Results

### Quantitative Information Transfer

**Figure 1:**
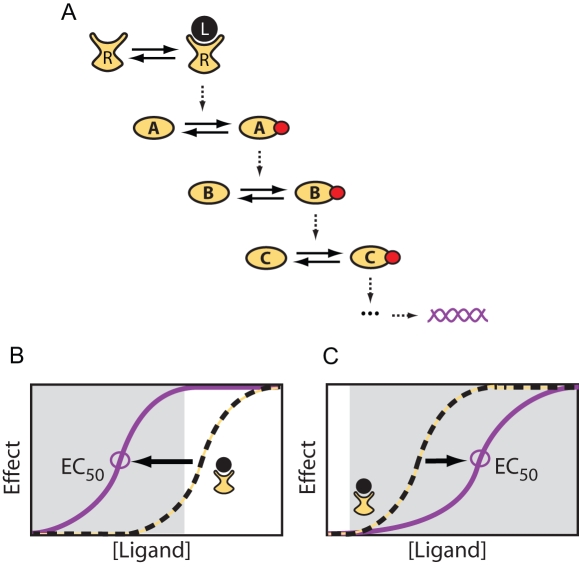
Dose response in signaling pathways. (A) A linear signaling pathway (L: ligand, R: receptor, A–C: signaling molecules, red dots: phosphate groups). (B) Dose response curves for a linear pathway. Saturation of a downstream pathway component leads to a shift of the physiological response curve (purple) to the left of the receptor occupancy curve (dashed black). Sensitivity at low stimulus doses is increased but the dynamic range (gray area) is reduced. (C) Dose response curves for a nonlinear pathway. Feedback or feed forward regulation can extend the dynamic range of the system (purple curve) and produce responses with an EC_50_ value displaced to the right of the receptor K_d_.

In the simplest scenario of a linear signaling pathway subject to a sustained stimulus (Figure 1A), quantitative information about the dose of the stimulus can only be transmitted as the activity level of the signaling proteins that make up the pathway. We refer to this mode of signal transduction as “amplitude encoding” because information about the stimulus is contained in the amplitude of the propagated signal [8]. For linear pathways, the dynamic range, (i.e., the range of stimulus levels to which the pathway can respond in a dose-dependent manner) is limited when the activity of a pathway component becomes saturated. Often, a downstream component saturates before the receptor. Thus the agonist concentration required to achieve the maximum downstream response may be less than the concentration needed to saturate the receptors, causing the dose-response curve of the pathway to shift to the left of the receptor-occupancy curve (Figure 1B). Pharmacologists refer to this phenomenon as “amplification” or “receptor reserve”.

Signaling networks are rarely linear, however, and often include combinations of feedback and feed-forward loops that positively and negatively regulate pathway activity. Among other things, these regulatory mechanisms allow signaling systems to respond transiently (adapt) to a persistent stimulus. We show that transient pathway activation provides the possibility of “dose-to-duration” encoding. That is, information about the stimulus concentration is transduced as the duration of the propagated signal rather than the amplitude. We demonstrate that an advantage of dose-to-duration encoding is that it provides a mechanism for increasing the dynamic range of signaling systems by allowing them to respond in a dose-dependent manner even after pathway components have become saturated, even at agonist concentrations that saturate the receptor (Figure 1C).

In the following sections, we analyze dose-to-duration encoding as a means for relaying quantitative information about the extracellular environment and discuss simple pathway architectures capable of carrying out this conversion. The key information transfer in this strategy occurs during the transient activation of pathway components rather than through their steady state levels of activation [9]. Next, we present data for MAP kinase activity that demonstrates dose-to-duration encoding is used in the pheromone response pathway of yeast. Finally, we present a mathematical model of the pathway based on the mechanisms discussed here that is consistent with experimental observations.

### Encoding Information as Signal Duration

**Figure 2:**
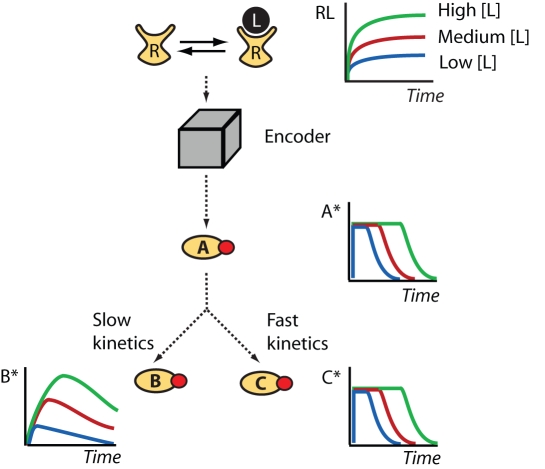
Dose-to-duration encoding. The receptor occupancy level is proportional to the ligand concentration. An encoder transforms receptor occupancy (RL) into the duration of protein A activity (A*). A* activates two downstream proteins, B and C. Because of its slow activation kinetics, B acts as an integrator transforming the duration of A activity into the amplitude of B activity (B*). Protein C has fast kinetics and therefore its activity level (C*) follows A* and information continues to be transmitted as signal duration.

Dose-to-duration encoding requires the propagated signal to act transiently. That is, at least one component of the pathway must return to its pre-stimulus level on a time-scale significantly shorter than that of the physiological response. This transient activity can result from the stimulus itself acting transiently or arise because the pathway contains regulatory elements that convert a sustained input into a transient output. The first case is commonly observed in inter-cellular signaling, where the duration of pathway activity often is regulated by the slow degradation or elimination of the agonist (e. g., reuptake of a neurotransmitter) [10],[11]. We focus on the second case, that is, adaptive systems that possess the ability to convert the intensity of the input signal into duration of the output signal in the presence of a persistent stimulus. Figure 2 shows schematically how dose-to-duration encoding works. In this example, a fixed agonist concentration quickly activates receptors in the plasma membrane. The steady-state level of active receptor (input) causes the activation of a signaling module (“encoder”, grey box) that generates a transient activation of the signaling protein A. We use an asterisk to denote the active form of a protein (e.g., A*). The output of the encoding module is a signal of constant amplitude but dose-dependent duration (A* in Figure 2). At each stimulus dose, the amplitude of A* rapidly saturates, but information about the level of receptor occupancy is preserved in the duration of the A* signal. Mechanisms capable of such dose-duration transformations are the subject of the next section.

Figure 2 shows two possible scenarios for how A* activates its downstream targets. In the first scenario, species B is slowly activated by A*. This causes the activity of B (B*) to increase during the entire period of A's transient activation. If the kinetics for the deactivation of B also are slow, B activity remains elevated for a significant amount of time after the A* has returned to its basal level. In this case B effectively works as a decoder, transforming the duration of A activity into the amplitude of B activity. In other words, slow kinetics makes B an integrator capable of measuring how long the upstream signal has been on. In the second scenario depicted in Figure 2, species C has fast activation and deactivation kinetics. As a result, the C* concentration closely mimics the behavior of A*, reaching a quasi-equilibrium level soon after the signal is received and rapidly returning to pre-stimulation levels once A activity ceases. In this case, quantitative information about the stimulus is preserved even when C* is saturated because it is encoded as signal duration. We note that dose-to-duration encoding does not place restrictions on what types of responses a cell can initiate. For example, positive feedback acting downstream of either components B or C can be used to convert transient pathway activation into a permanent developmental switch [12].

### Simple Architectures That Function as Duration Encoders

**Figure 3:**
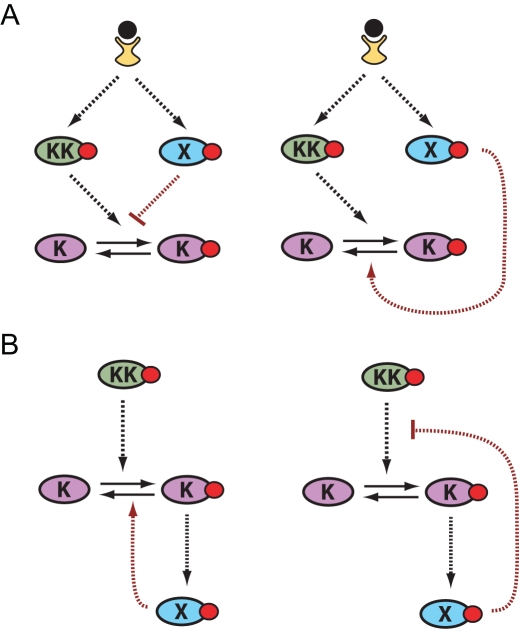
Pathway architectures that convert stimulus dose to signal duration. (A) Feed-forward and (B) negative feedback encoding modules (KK: Kinase-Kinase, K: Kinase, X: Phosphatase). Shown are cases of negative regulation operating by inhibiting activation (left diagrams) or promoting deactivation (right diagrams).

In this section we discuss mechanisms that can achieve dose-to-duration encoding. As previously mentioned, we are focusing on cases involving a sustained input, and therefore need to consider systems capable of adaptation or desensitization. In order to work as a dose-to-duration transducer, the duration of the output has to increase with the concentration of the stimulus. As we illustrate below, this is not a general property of adaptive systems. Figure 3 shows a number of architectures capable of performing the dose-to-duration transformation. The two pathway architectures depicted in Figure 3A consist of incoherent feed-forward loops [13] in which the upstream stimulus activates both a positive and negative regulator of the signaling protein K. For the system to show transient activity, negative regulation must occur on a slower time scale than the activation rate of K. As shown in the figure, this can be achieved if the negative regulation is mediated by an intermediate species X. This species can operate either by inhibiting activation of K by KK or by promoting deactivation of K. This type of architecture occurs in ERK signaling networks in which agonists, such as epidermal growth factor, causes transient extracellular signal-regulated kinase (ERK) activation by triggering rapid Ras activation followed by slow recruitment of its negative regulator, Ras GTP-ase regulating protein (Ras-GAP), to the membrane [14].

Figure 3B shows two simple pathway architectures involving negative feedback loops that can exhibit adaptive behavior. In these examples, the signaling molecule activates its own negative regulator. In the first case, the negative regulator X increases the deactivation rate of K and in the second case X decreases the activation rate of K. Both strategies produce qualitatively similar behavior. Similar to the case of feed forward regulation, adaptive behavior in these systems requires the negative feedback to operate on a slower time scale than that of activation of K. This type of architecture plays a role in the regulation of ERK signaling by the enzymatic activation of members of the MAPK phosphatase group (MKP's) [15]–[17], and in the regulation of cytokine signaling by the induction of suppressor of cytokine signaling (SOCS) proteins [18].

**Figure 4:**
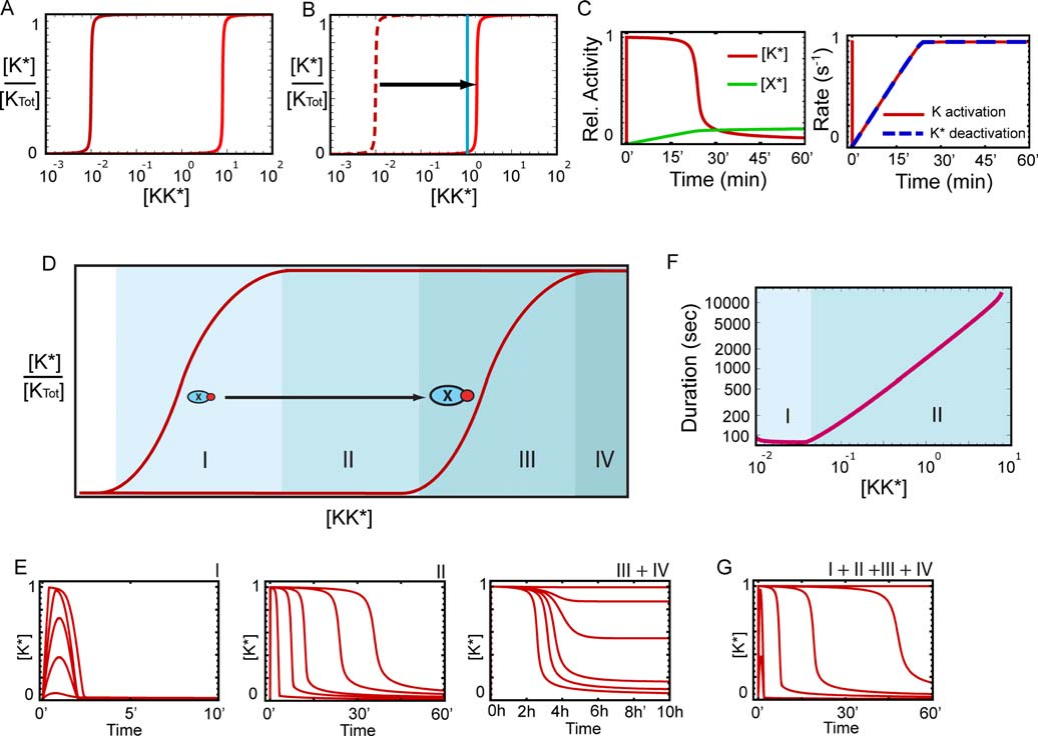
Dose-to-duration encoding by negative feedback. The units of concentration are arbitrary and time is measured in minutes. The responses are normalized to the total concentration of the respective proteins. (A) Response curves for species K shown in the absence of the negative regulator X (left curve) and in the presence of maximal X activity (right curve). (B) Displacement of the response curve during a signaling event. Blue curve: KK* level used to generate the curves in (C). (C) Time profiles of [K*] (red curve) and [X*] (green curve, left panel) and the activation rate of K (red curve) and deactivation rate of K* (dashed blue curve, right). Note that after an initial spike in the activation rate, the two rates roughly equalize satisfying the quasi-equilibrium condition. The system adapts when the activation rate can no longer increase and compensate for the increasing X activity. (D) An expanded version of the response curves shown in (A) indicating the four possible operational regimes. (E) Time courses of K activity illustrating the four operational regimes. (F) Signal duration (defined as time between half maxima) vs. KK* concentration. Regimes I and II are shown. (G) Same as (E) except the different regimes are now shown on the same graph.

We focus primarily on the negative feedback system depicted by the left diagram in Figure 3B, but the results that follow easily generalize to the other architectures. The equations that describe this model are given in the Methods. To understand how this system performs the dose-to-duration transformation, it is helpful to consider the steady-state response curve of K as a function of the activity of the upstream component KK in the absence and presence of the negative regulator X. In Figure 4A, the left curve corresponds to the case in which X has been deleted. When present, the effect of the negative regulator X is to shift the signal-response curve to higher active KK concentrations. Accordingly, the right curve shown in Figure 4A corresponds to the case in which X is maximally activated.

The response of the system now can be understood by considering how the signal-response curve shifts in time (Figure 4B). This approach is valid because the requirement of slow negative feedback implies that the K and K* concentrations are in quasi-equilibrium with respect to the current X* concentration. Figure 4C (left) shows time series for an example in which the level of KK* is given by the blue vertical line in Figure 4B. Upon activation, KK* quickly drives the level of K activity to the steady-state value expected in the absence of the negative regulator X, which for this example corresponds to full activation. Subsequent to the initial rapid rise of K*, the activation and deactivation rates roughly balance (Figure 4C, right) and the relative ratios of K and K* remain in a quasi-equilibrium determined by the current level of X*. As the level of X* slowly increases, the signal-response curve gradually shifts to the right (Figure 4B), and eventually, the EC_50_for K activation moves beyond the available concentration of KK. At this point the concentration of X* is such that the stimulus cannot counteract the level of negative regulation and K* activity returns to near basal levels (Figure 4C, left).

The two properties required for the dose-to-duration transformation are that (i) the activation rate of K is proportional to the stimulus concentration (KK* concentration in the model under consideration) and (ii) the kinetics of the negative regulator are slow. It is also important that the negative regulator induces a reversible change in K rather than on irreversible change, such as degradation or irreversible desensitization. Under these conditions, by slowly increasing the deactivation rate of K*, the system is actually “measuring” the activation rate of K rather than the K* concentration. The readout is the time necessary to produce enough X* to counteract the stimulated activation rate of K and bring K* back to basal levels. Importantly, this approach can potentially be used to measure stimulus concentrations much higher than those that would saturate pathway activity in the absence of negative feedback. *In other words, by exploiting the time-dependent properties of the system, signaling pathways can increase their dynamic range.*

The dose-to-duration transformation described above occurs most efficiently when the activity level of the signaling component involved in the transformation is saturated. More generally, there is a repertoire of four operational regimes available to the adaptive system. These are summarized in Figure 4D and 4E. Figure 4D again shows the steady state response curves in the absence (left) and presence (right) of the negative regulator. In this figure, the graph has been expanded for illustrative purposes. The four shaded regions correspond to the different operational regimes. The first regime corresponds to low stimulus concentrations. When the stimulus strength increases within this regime, the response of the system consists of transient peaks of increasing amplitude but roughly the same duration (Figure 4E, left). For each stimulus, the peak amplitude can be approximately determined by the signal-response curve in the absence of negative regulation. Increased upstream K activity increases the rate at which X is activated, and hence only a relatively weak dependence of the signal duration on the stimulus dose is observed.

Regime II arises when the stimulus strength is sufficient to saturate K activity. This is the regime in which the dose-to-duration transformation occurs (Figure 4E, center). Figure 4F presents the relationship between signal duration (defined as time between half-maxima) and stimulus concentration in Regimes I and II. In Regime III, the stimulus level is high enough so that the negative regulator is no longer able to counteract the induced activation rate of K, even when X is maximally activated. In this regime the system begins to lose its ability to adapt (Figure 4E, right). If the stimulus level increases even further, the system operates in Regime IV and adaptation no longer occurs (Figure 4E, right). In this regime, a sustained input produces a sustained output. Therefore, this pathway architecture is capable of acting as a switch; at low stimulus dose the response is transient, whereas at high levels the response becomes sustained. To illustrate how the transition between these regimes occurs, Figure 4G shows characteristic time series from each regime on the same graph. Physiological conditions and kinetic properties of signaling pathways may constrain some systems to operate in a subset of the theoretically possible regimes.

The minimalistic systems described here are intended to illustrate some of the mechanisms available to signaling networks for producing dose-to-duration encoding. Although very simple, they are useful for understanding the behavior of more complex architectures. The addition of more pathway components would not change the underlying operating principles of dose-to-duration encoding. In fact, additional components can be used to generate more robust responses and provide more opportunities to fine-tune the input-output relations of the pathway.

### Dynamic Regulation of the Receptor Allows Signaling beyond Saturation

**Figure 5:**
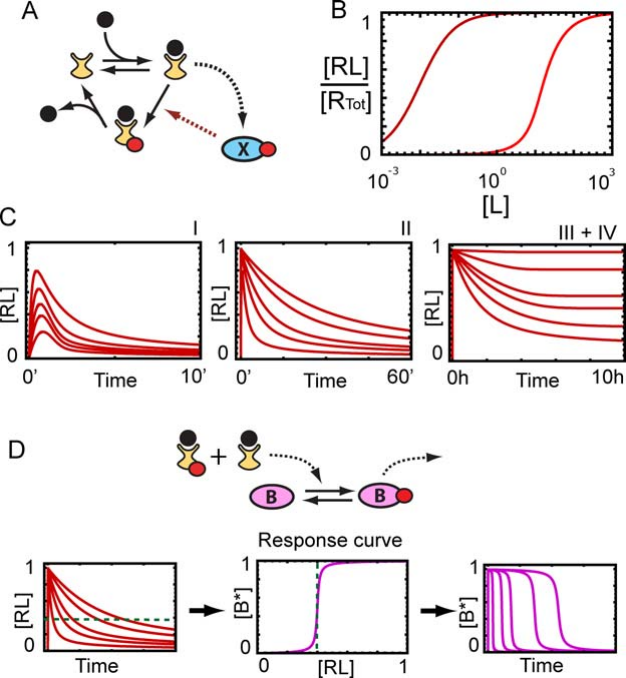
Feedback regulation of receptor affinity. The units are the same as in Figure 4. (A) The active receptor activates species X, which in turn phosphorylates the ligand-bound receptor, decreasing the affinity of the receptor for the ligand. (B) Receptor occupancy curves in the absence and presence of active X. (C) Time courses for the ligand-bound receptor corresponding to the four operational regimes shown in Figure 4. (D) The temporal profiles from Regime II in (C) are used as input signals for species B. The switch-like response of B converts the input signal into a square-pulse output signal.

When operated in Regime II, the temporal profile of K* resembles a square pulse (Figure 4C). This is because the signal-response characteristics of the system in the absence of the negative regulator were taken to be switch-like. Therefore, it is important to study how the dose-to-duration transformation is affected when this assumption is relaxed. We start by observing that the switch-like signal-response curves result from the small values of the Michaelis constants used in the reaction rates (k_1m_, k_2m_, and k_3m_ in Equation1), which means that the reaction rates saturate at low substrate levels. In the opposite extreme, the activation rates operate far from saturation. In this case, the catalytic reactions can be described in terms of mass action kinetics. For the system to efficiently adapt, a relatively steep dose-response curve is still required. This can be achieved by manipulating the parameters involved in the negative regulation. For such cases, the system's response to a sustained stimulus is no longer a square pulse, but shows a more gradual decay in time (cf. Figure 5C, middle). However, as we show next, the length of time required for the signal to decline below a given threshold still depends on the strength of the stimulus, and therefore the stimulus concentration can still be encoded as signal duration.

The scenario discussed above is of particular interest because it applies to a situation in which the negative feedback loop acts at the level of the receptor. Figure 5A shows a schematic diagram of a model in which the ligand-bound receptor activates a negative pathway regulator X. The protein X inhibits the pathway by modifying the ligand-bound receptor (phosphorylation in this example) and decreasing its affinity for the ligand (Figure 5A). Equations 2–4 of the Methods provide a mathematical description of this model. Figure 5B shows the steady-state receptor occupancy curves in the absence and presence of the negative regulator X. Temporal responses of the ligated receptor concentration for the four operational regimes are shown in Figure 5C. Note that while these time series do not have square pulse shapes, dose-to-duration encoding is still possible, because higher ligand levels cause active receptors to persist for longer times (Figure 5C, middle). Furthermore, a square-pulse activity profile is easily generated if the pathway contains a downstream component with switch-like signal-response characteristics. As shown in Figure 5D, the pathway component B measures how long receptor occupancy remains above its activation threshold, thereby transforming the time series for receptor occupancy into a square-pulse of B activity.

An important consequence of this pathway architecture is that it allows for “signaling beyond saturation”. That is, the system responds in a dose-dependent manner to ligand concentrations higher than required to saturate the receptor (Figure 1C). In other words, the dissociation constant of the receptor can be dynamically modulated and exploited to expand the dynamic range of the signaling pathway.

### Dose-to-Duration Encoding in the Yeast Pheromone Pathway

**Figure 6:**
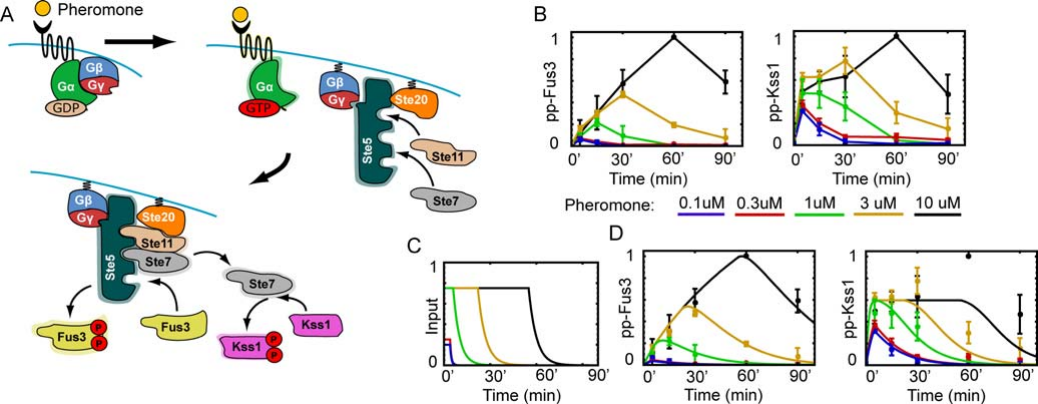
The yeast pheromone response pathway. (A) Schematic diagram of the pathway. (B) Experimentally obtained time series for the dually-phosphorylated (active) forms of the MAP kinases Fus3 and Kss1 normalized to the maximum response. The different colored data points correspond to different pheromone concentrations. (C) Proposed upstream Ste7 signals. (D) Comparison of experimental data (circles) and model output (curves) for time courses of Fus3 and Kss1 activity. The model results were generated using the Ste7 signals shown in (C) as input.

The mating response pathway of yeast mediates the organism's response to pheromone secreted into the medium by cells of the appropriate mating type. When bound with pheromone, a specific G-protein coupled receptor activates its cognate G protein causing the dissociation of the α and βγ subunits. The βγ complex then recruits the scaffold protein Ste5 to the membrane, which in turn recruits and activates a signaling cascade composed of Ste20 (MAP4K), Ste11 (MAP3K), Ste7 (MAP2K), and the MAP kinases Fus3 and Kss1 (Figure 6A, for a review see [3]). The developmental response initiated by yeast depends critically on the pheromone concentration. In the presence of very low levels or no pheromone, cells continue to grow and divide normally. At intermediate levels of pheromone, the cells become elongated and are capable of chemotropic growth towards a pheromone gradient. High levels of pheromone produce a bona fide mating response, involving cell division arrest and the emergence of mating projections [3],[5],[6]. We recently published an experimental study demonstrating that the scaffold protein Ste5 slows the activation rate of the MAP kinase Fus3 and that this slow activation underlies the developmental switch from chemotropic growth to mating [7]. In this section we present a mathematical analysis of the temporal profiles of MAP kinase activity measured as a part of our previous investigation. Our analysis suggests that the mating response pathway is using dose-to-duration encoding to relay information about the extracellular pheromone concentration.

Figure 6B shows time course data for active (dually-phosphorylated ) Fus3 and Kss1 as measured by immunoblotting for wild type cells in response to different pheromone concentrations (see [7] and methods for details of the experimental methods). The transition from chemotropic growth to mating occurs between 3 and 10 µM, where there is a large increase in Fus3 activity [7]. Note the qualitative similarity between the experimental results and the graphs in Figure 2 (compare pp-Fus3 to B* and pp-Kss1 to C*). The roughly dose-independent rate (slope) for Fus3 phosphorylation suggests that its activation rate is saturated. This behavior is consistent with the level of upstream kinase activity being independent of the pheromone dose, whereas the duration of this activity is dose-dependent. On the other hand Kss1 shows fast kinetics. Note that for high pheromone concentrations (10 µM), Kss1 seems to undergo two stages of phosphorylation with a second increase in phosphorylation starting around 30 min after exposure. If we disregard this second increase in Kss1 activity (see Discussion), then by virtue of its fast kinetics, Kss1 phosphorylation mirrors the upstream signal dynamics. Furthermore, it appears from the data that for the doses measured, Kss1 operates in Regimes I and II (and perhaps III) of Figure 4. These observations when combined with the very good correlation between the duration of Kss1 and Fus3 activity, suggest that Fus3 and Kss1 phosphorylation are driven by an upstream signal in which the pheromone dose has been converted to signal duration.

To test the idea of dose-to-duration encoding, we sought to establish a single upstream input profile capable of reproducing the experimental results for both Kss1 and Fus3. Specifically, we looked for a signal profile s(t) that when used as input to the equations for Fus3 activation (Equation 7) and Kss1 activation (Equation 8) generates the Fus3 and Kss1 activity time series shown in the left and right panels, respectively, of Figure 6B. The analysis produced the input signals and MAP kinase profiles shown in Figure 6C and 6D, respectively. The excellent agreement between the experimental data and model output provides strong evidence in support of dose-to-duration encoding by the pheromone response pathway.

In principle, any of the encoding mechanisms discussed in the previous sections can produce temporal profiles similar to Figure 6C (see Figures 4 and 5), and there are several potential candidates for the negative feedback loop that mediates the dose-to-duration transformation in the mating pathway. These include transcriptional induction of either the RGS protein Sst2, which increases the rate at which the Gα subunit hydrolyzes GTP [19], or the protease Bar1, which degrades pheromone [20],[21]. Note that transcriptional induction takes 30 minutes or more. Because pathway deactivation occurs within 30 min at low pheromone concentrations, it is likely that feedback loops involving protein modifications also play a role in the dose-to-duration transformation. Furthermore, because the MAP kinases respond in a dose-dependent manner at pheromone concentrations significantly higher than the reported receptor K_d_ value of 5–15 nM [22],[23], it is plausible that dose-to-duration encoding involves feedback regulation of the receptor. This is not the only possibility and any target of feedback regulation at the level of the MAP2K Ste7 or above would work equally well (see Discussion).

**Figure 7:**
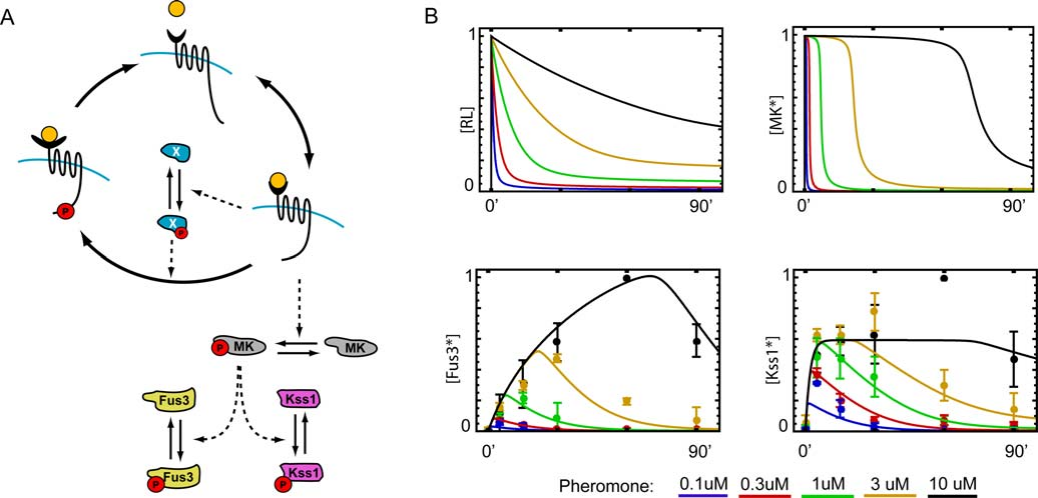
A model for dose-to-duration encoding in the pheromone response pathway. (A) Receptor affinity is feedback-regulated by species X. The signal is converted into a square pulse by the intermediate kinase MK (e.g., Ste20, Ste11, or Ste7), which also activates the MAP kinases Fus3 and Kss1. (B) Time courses of signal activity at different stages of the pathway: receptor occupancy (top left) and [MK*] (top right), and Fus3 and Kss1 activity (bottom left and right, respectively).

With the above considerations in mind, we developed a mathematical model to investigate the scenario in which the negative feedback loop acts on the receptor. Figure 7A shows a schematic diagram of the system and the shape of the propagated signal at each level of the pathway. The model is described by Equations 3–5 and 7–9 of the Methods. As discussed in the previous section, because the negative feedback acts on the receptor, it is necessary to incorporate an intermediate step (MK in Figure 7) to transform the propagated signal into a square-pulse. Any of the upstream kinases (Ste20, Ste11 or Ste7) are capable of performing this transformation. Figure 7B shows the predicted upstream activation profile (compare with Figure 6C) and the MAP kinase activation profiles produced by the models compared with the experimental results. If we again disregard the second increase in Kss1 activity at high pheromone concentrations, the correspondence between the model results and experimental data is striking, especially considering the simplicity of the model. We note that this agreement does not prove the validity of the model, but demonstrates that the mechanisms discussed above are consistent with the experimental data. The model also provides an important guide for future experimental work.

## Discussion

It is widely accepted that signaling pathways are capable of transmitting quantitative information about their surrounding environment. While the importance of transient versus sustained signaling has been recognized for some time [24]–[26], most previous investigations have focused on information transfer using amplitude encoding without considering the temporal aspects of signal transduction [8],[9]. Here we demonstrate that dose-to-duration encoding provides cells with an alternative mechanism for processing and transmitting quantitative information about their surrounding environment. The ability of signaling pathways to convert stimulus strength into signal duration results directly from the nonlinear nature of these systems and emphasizes the importance of considering the dynamic properties of signaling pathways when characterizing their behavior. Taken together, our computational and experimental results suggest that dose-to-duration encoding occurs in the pheromone response pathway of yeast and underlies the developmental switch from chemotropic growth to mating.

One important advantage of dose-to-duration encoding is that it has the potential to increase the dynamic range of signaling pathways. One way this can occur is if feedback regulation allosterically modifies the receptor's affinity for the ligand. Dynamically regulating the K_d_ of the receptor has the interesting effect of shifting the EC_50_ of the cellular response to the right of the receptor occupancy curve (Figure 1C). Depending on the response of downstream components, the dose-response curve for the system is not only shifted but also stretched. This highlights the important point that receptor occupancy curves are potentially time-dependent quantities and need to be interpreted with care. Interestingly, K_d_ values determined in vitro or in reconstituted systems usually differ from those obtained in vivo, and this discrepancy is often attributed to an abnormal conformation of the receptor in the artificial environment. The analysis presented here suggests that even when there is correspondence between the microenvironment of an in vitro experiment and the macroenvironment of a cell culture, the results of ligand binding assays might differ due to dynamic regulation of the receptor in vivo. For example, this could happen if a downstream element of the signaling pathway has been disrupted in the in vitro experiment, thereby breaking the negative feedback loop. Interactions between receptors, in particular G-protein coupled receptors (GPCRs), and cytosolic proteins have been shown to affect receptor-ligand affinity [27]. Most GPCR's are known to undergo biochemical modifications, such as phosphorylation, and to interact with a number of signaling proteins, including G proteins, arrestins, kinases, RGS proteins, and to form oligomers, all of which could affect affinity for the ligand. Therefore dynamic regulation of a receptor as a mechanism for dose-to-duration encoding seems quite plausible.

Dose-to-duration encoding may also provide a more robust transmission mechanism than amplitude-encoding in multilevel networks. This is because accurate transfer of information using amplitude encoding requires that the input-output characteristics of the individual components be well matched [8]. Note that dose-to-duration encoding does not have to function throughout the whole pathway. It is likely that multiple information processing strategies coexist at different levels (or even under different conditions) in a single pathway. In fact, the use of multiple information processing strategies may provide signaling networks with more flexibility when responding to changing environmental conditions. Another potential advantage of dose-to-duration encoding arises from the need to prevent spurious activation of pathways that share components. Recently we proposed “kinetic insulation” [28] as a strategy for achieving pathway specificity. Kinetic insulation relies solely on the temporal profiles of the propagated signals to ensure signal fidelity. It requires that at least one of the pathways responds transiently. Because signal duration is a natural strategy for pathways with transient activity to encode information, signaling systems with shared components are potential candidates for dose-to-duration encoding. Consistent with these ideas, the yeast pheromone response pathway contains several signaling proteins (e.g., Ste11 and Ste7) that are known to also participate in the hyper-osmotic shock [29] and filamentous growth [30],[31] pathways.

Our modeling results and experimental data provide compelling evidence for dose-to-duration encoding by the yeast pheromone response pathway. A key question is then what is the molecular mechanism responsible for transducing stimulus dose into signal duration? We have demonstrated that a scenario in which feedback regulation acts at the level of the receptor is consistent with our experimental data for MAP kinase activity. Our motivation for considering such a mechanism came from data suggesting that yeast continue to respond in a dose-dependent manner to pheromone concentrations well beyond the reported value for the receptor dissociation constant. As we have shown, by dynamically altering the affinity of the receptor for pheromone, our model provides an explanation for this phenomenon of signaling beyond saturation. Modulation of the receptor affinity in yeast might occur by interactions with other receptors (receptor dimers) [32], the G-protein [27], or the RGS protein Sst2 [33]. Similarly, affinity could be altered through receptor phosphorylation or ubiquitination [34]. GTP-dependent changes in the pheromone receptor affinity attributed to the interaction with the G protein have been reported [27]. Although the physiological relevance of this effect has not been clearly established in yeast, this is an example of a phenomenon observed for many mammalian GPCR's in vitro.

We note that interpreting dose-response data for the pheromone pathway is complicated by the presence of the protease Bar1 [20],[21]. In fact, a mechanism based solely on Bar1 degradation of pheromone is in theory sufficient to achieve the dose-to-duration transformation. However, our recent experiments performed in a *bar1Δ* mutant show cells responding to lower pheromone doses, but with time courses of MAPK activity that are consistent with dose-to-duration encoding (Supplemental Data [7]). These results argue against a mechanism involving Bar1 alone. It should be emphasized that dose-to-duration encoding does not require the negative feedback to act at the level of the receptor. For example, induction of the negative regulator Sst2 [35], feedback phosphorylation of an upstream pathway component [36], or receptor endocytosis could also accomplish this transformation, although they would not account for the observed shift in the EC_50_. Thus, it is clear more work is necessary to unambiguously identify the mechanisms responsible for information transfer in the pheromone response pathway. However, the remarkable agreement between our modeling results and experimental data offers strong evidence in support of dose-to-duration encoding and provides a foundation for interpreting future experimental results.

Interestingly, the combination of fast and slow kinetics exhibited by the two MAP kinases, Kss1 and Fus3, has the potential to form a feed-forward adaptive system. It has been demonstrated that pheromone-induced degradation of the transcriptional activator Ste12 requires Fus3, but not Kss1 [37]. Ste12 might also play a role in generating the second peak of Kss1 activity observed at high pheromone concentrations, a possibility that we are now investigating. In the absence of pheromone, Kss1 acts as a transcriptional repressor by forming a complex with Ste12 and the proteins Dig1 and Dig2 (also known as Rst1/2) [38]. It is possible that pheromone-stimulated release of Kss1 from this complex [39] generates a second pool of Kss1 and this pool is responsible for the second peak of activity. However, at this point we cannot rule out alternative explanations including transcriptional induction, re-localization, or positive feedback.

Dose-to-duration encoding is not restricted to yeast. For example the intensity of light (number of photons) impinging on photoreceptors in rod cells is encoded as the duration of G protein-mediated activity of the pathway [40]. It has been shown recently that the RGS protein RGS9 plays a particularly important role in determining the duration of the signal [41]. Furthermore, switches based on transient versus sustained signals, like the ones arising from transitions between the regimes of Figure 4, have been proposed to underlie cell fate decision process in a number of systems [14],[26],[42],[43]. The recent discovery that different temporal profiles of IκB kinase (IKK) activity in the NF-kB signaling module selectively activate different groups of target genes, further supports the notion that dose-to-duration encoding plays a significant regulatory role in determining cellular responses. In this case, stimulation of murine embryonic fibroblasts with tumor necrosis factor-α produces a short transient peak of IKK activity whereas stimulation with polysaccharides results in a slower and more sustained IKK response [44],[45]. The fact that each profile affects the expression of different groups of genes illustrates how the temporal dynamics of a signaling pathway can play a role in determining pathway specificity.

Finally, it is remarkable that the simple pathway architectures considered here can generate such a variety of responses depending on the strength of the stimulus (Figure 4). These systems not only function as amplitude and dose-to-duration encoders, but also can act as biochemical switches that transition from transient to sustained outputs potentially generating different physiological responses [26],[42],[46]. Typical signaling pathways involve multiple levels of regulation that in general should lead to even more complex behavior. Our results demonstrate how quantitative measurements of the temporal patterns of pathway activity when combined with mathematical modeling can be used to discover the design principles upon which signaling networks operate and decipher the code used by these systems to transmit information.

## Methods

### Model Equations

In this section we describe the mathematical models used to generate the results presented in the figures. All the differential equations were solved using Mathematica by Wolfram Research. The model depicted in Figure 3B (left), in which the mechanism for pathway adaptation involves a negative feedback loop that increases the deactivation rate of K*, is described by the following two equations:
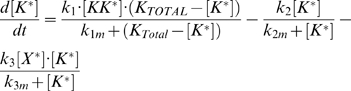
(1)

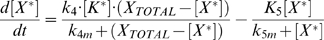
(2)Where [K]_Total_ = [K*]+[K] and X_Total_ = [X*]+[X]. The parameter values used to generate the results shown in Figure 4 are (in arbitrary units): k_1_ = 1, k_1m_ = 10^−2^, k_2_ = 10^−2^, k_2m_ = 10^−2^, k_3_ = 8, k_3m_ = 10^−2^, k_4_ = 10^−4^, k_4m_ = 10^−1^, k_5_ = 5 10^−6^, k_5m_ = 1. The curves in this figure correspond to s values of: 10^−2^, 2 10^−2^, 3 10^−2^, 4 10^−2^, 6 10^−2^ (region I), 10^−1^, 3 10^−1^, 5 10^−1^, 1, 1.5 (region II), and 6.0, 7.0, 7.5, 8.0, 8.5, 20.0 (region III+IV).

For the model in which the negative feedback acts on the receptor, the equations are:
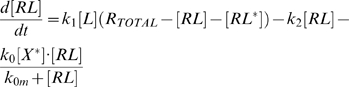
(3)

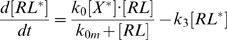
(4)

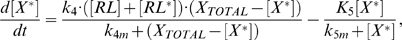
(5)where R_Total_ = [R]+[RL]+[RL*]. For simplicity, ligand release and receptor de-phosphorylation are taken to occur in a single step. This simplification does not affect the results provided both biochemical steps are not rate limiting. Even if this separation of times scales does not exist, we do not expect a more detailed model that separates these events to produce qualitatively different behavior.

To transform the transient response in Figure 5C into a square pulse the following equation for B* was used
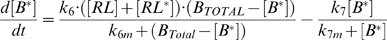
(6)The parameters used to produce the results shown in Figure 5 are k_1_ = 1, k_2_ = 10^−2^, k_3_ = 80, k_4_ = 1 10^−4^, k_4m_ = 10^−1^, k_5_ = 5 10^−6^, k_5m_ = 1, k_0_ = 10, k_0m_ = 10^−1^, k_6_ = 10, k_6m_ = 10^−2^, k_7_ = 4, k_7m_ = 10^−2^. The curves correspond to s values of: 1 10^−2^, 2 10^−2^, 3 10^−2^, 5 10^−2^, 1 10^−1^ (region I), 10^−1^, 3 10^−1^, 5 10^−1^, 1, 1.5 (region II), and 6, 10, 15, 20, 50, 500 (region III+IV).

The equations used to model the kinetics of Fus3 and Kss1 activation are
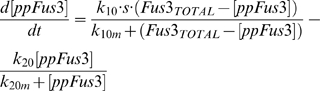
(7)

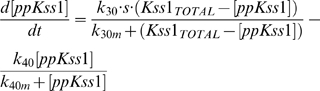
(8)respectively, where Fus3_Total_ = [ppFus3]+[Fus3], Kss1_Total_ = [ppKss1]+[Kss1]. The parameters used to produce the results shown in Figure 6 are: k_10_ = 5.53 10^−4^, k_10m_ = 3.75 10^−2^, k_20_ = 3.25 10^−4^, k_20m_ = 3 10^−1^, k_30_ = 2.55 10^−2^, k_30m_ = 1, k_40_ = 2.5 10^−3^, k_40m_ = 2. The input signals consist of a square pulse of duration *tpulse* followed by an exponential decay (i.e., signal = S for time<tpulse, and signal = S e^−(time-tpulse)/λ^ for time>tpulse). The signal parameter for each concentration were as follows: S = 0.2, 0.25, 0.75, 0.75, 0.75, tpulse = 55′, 22′, 6′, 4′, 4′, and λ = (50, 50, 250, 300, 300)×3600 min.

The full model depicted in Figure 7 is described by Equations 3–5 and 7–8 above, in which s has to be replaced by [MK*]. The following equation describes the dynamics of MK*:
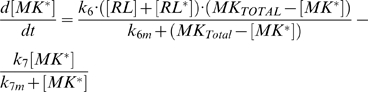
(9)Here MK_Total_ = [MK*]+[MK]. The parameters used to produce Figure 7 are (arbitrary units): k_1_ = 2, k_2_ = 3 10^−2^, k_3_ = 1.9 10^2^, k_4_ = 1 10^−4^, k_4m_ = 3 10^−2^, k_5_ = 8.5 10^−8^, k_5m_ = 2.5 10^−2^, k_0_ = 6.6 10^1^, k_0m_ = 5.1 10^−2^, k_10_ = 4.1 10^−4^, k_10m_ = 4.4 10^−4^, k_20_ = 5.9 10^−4^, k_20m_ = 4.6 10^−1^, k_30_ = 2.8 10^−2^, k_30m_ = 2.6, k_40_ = 1.15 10^−3^, k_40m_ = 3.8 10^−1^, k_6_ = 3.2 k_6m_ = 4.9 10^−4^, k_7_ = 1.7, k_7m_ = 3.3 10^−1^.

### Parameter Selection

As described in [47] the signaling modules presented above are capable of producing adaptive behavior for a wide range of parameter values. The main condition that must be met is that activation occurs on a fast time scale as compared to the feedback inhibition. The parameters for the examples used to illustrate dose-to-duration encoding were selected to comply with this requirement. The parameters for Figure 6 were tuned manually to generate a good fit to the data. However, the number of experimental points leaves significant leeway for the exact shape of the decay phase of the input signal. The parameters used to generate the curves for Figure 7 were obtained using a Monte Carlo algorithm. The values of the rate constants associated with ligand binding and dissociation in the absence of feedback regulation were fixed to reflect a K_d_ value of 15 nM [23].

### Experimental Methods

Immunoblot data for kinases Fus3 and Kss1 were obtained from [7]. Briefly, BY4741 (MAT**a** *leu2Δ met15Δ his3Δ ura3Δ*) cells were grown using standard practices. Cell extracts (20 µg/lane) were resolved by 12% SDS-polyacrylamide gel electrophoresis and immunoblotting performed as described in [42]. Band intensity was quantified by scanning densitometry using ImageJ (National Institutes of Health).
